# PDGFBB facilitates tumorigenesis and malignancy of lung adenocarcinoma associated with PI3K-AKT/MAPK signaling

**DOI:** 10.1038/s41598-024-54801-7

**Published:** 2024-02-20

**Authors:** He Xiu-Ying, Zheng Yue-Xiang, Yang Hui-Si, Yu Hong-Zhou, Xia Qing-Jie, Wang Ting-Hua

**Affiliations:** 1 Department of Anesthesiology, West China Hospital, Sichuan University, Chengdu, China; 2 Institute of Neurological Disease, West China Hospital, Sichuan University, Chengdu, 610041 Sichuan China; 3 Laboratory Zoology Department, Institute of Neuroscience, Kunming Medical University, Kunming, China; 4 School of Integrated Traditional Chinese and Western Medicine, Southwest Medical University, Luzhou, China

**Keywords:** Cancer, Biomarkers, Lung cancer, Non-small-cell lung cancer

## Abstract

Lung adenocarcinoma (LUAD) remains one of the most aggressive tumors and the efficacy of conventional treatment has been bleak. Nowadays, gene-targeted therapy has become a new favorite in tumor therapy. Herein, we investigated the effect of platelet derived growth factor BB (PDGFBB) on LUAD. Firstly, PDGFBB was upregulated in LUAD patients and closely linked with poor survival. Furthermore, the expression of PDGFBB and PDGFRα/β in LUAD cells was higher than that in normal lung cells. By loss-of-function with herpes simplex virus (HSV)-PDGFi-shRNA, we found that PDGFBB knockdown caused a significant decrease in proliferation and migration, but evoked apoptosis of LUAD cells in vitro. Conversely, exogenous PDGFBB held adverse effect. Additionally, A549 cells with PDGFBB knockdown had a low probability of tumorigenesis in vivo. Moreover, PDGFBB knockdown restrained the growth of xenografts derived from normal A549 cells. Mechanistically, PDGFBB knockdown suppressed PI3K/AKT and Ras/MAPK signaling, while PDGFBB was the opposite. Therefore, we concluded that PDGFBB might facilitate the tumorigenesis and malignancy of LUAD through its functional downstream nodes—PI3K/AKT and Ras/MAPK signaling, which supported that PDGFBB could serve as a rational therapeutic target for LUAD.

## Introduction

Lung cancer is the most common cancer and responsible for cancer-related mortality^1^. Based on the histology, it was classified into adenocarcinoma, squamous carcinoma, large cell carcinoma and small cell lung cancer^2^. Among these subtypes, lung adenocarcinoma (LUAD) is the most common subtype of primary lung cancer with the highest 5-year survival rate^3^. However, lung adenocarcinoma remains one of the most aggressive tumors and is usually diagnosed with disseminated metastatic tumor at advanced stage^4,5^, leading to the bleak outcome in the surgical treatments^6,7^. In addition, the resistance from the conventional radiotherapies and chemotherapies also has become the biggest obstacle in the treatment of lung cancer^3^. Nowadays, a greater understanding of the oncogenes and anti-oncogenes provides a novel way for the cancer treatment^8^. Molecular-targeted therapy holds a bright future^9^.

The PDGFs contain of four different polypeptide chains (PDGF-A, -B, -C, and -D) encoded by different genes, which form homodimers or heterodimers, including PDGF-AA, -AB, -BB, -CC, and -DD, and belong to a family of peptide growth factors^10^. These factors are involved in cellular process through cell surface PDGFα and PDGFβ protein tyrosine kinase receptors (PDGFRα/β), and stimulate various cellular functions including growth, proliferation and differentiation^11^. In recent years, PDGFs are found to be upregulated in many types of cancers, such as gastrointestinal stromal tumor, glioma, chronic myelomonocytic leukemia, nonmelanoma skin cancer, prostate cancer, ovarian cancer, and non-small-cell lung cancer^12–16^. Moreover, the inhibitors for PDGFs remarkably improved the survival^11^. As a member of the PDGFs family, the effect of PDGFBB (*PDGFB*) on cancers has been widely concerned. Now tumor-derived PDGFBB has been reported to promote tumor growth, angiogenesis and extramedullary hematopoiesis in oligodendroglioma, high-grade glioma and breast cancer^17–21^.

In this study, through constructing herpes simplex virus carrying PDGFi-shRNA (HSV-PDGFi-shRNA) and PDGFBB recombinant protein, we further explored the role of PDGFBB in LUAD and the related mechanism, which will provide a reference for the gene-targeted therapy of lung adenocarcinoma.

## Results

### PDGFBB was upregulated and associated with poor outcomes in lung adenocarcinoma patients

**Figure 1:**
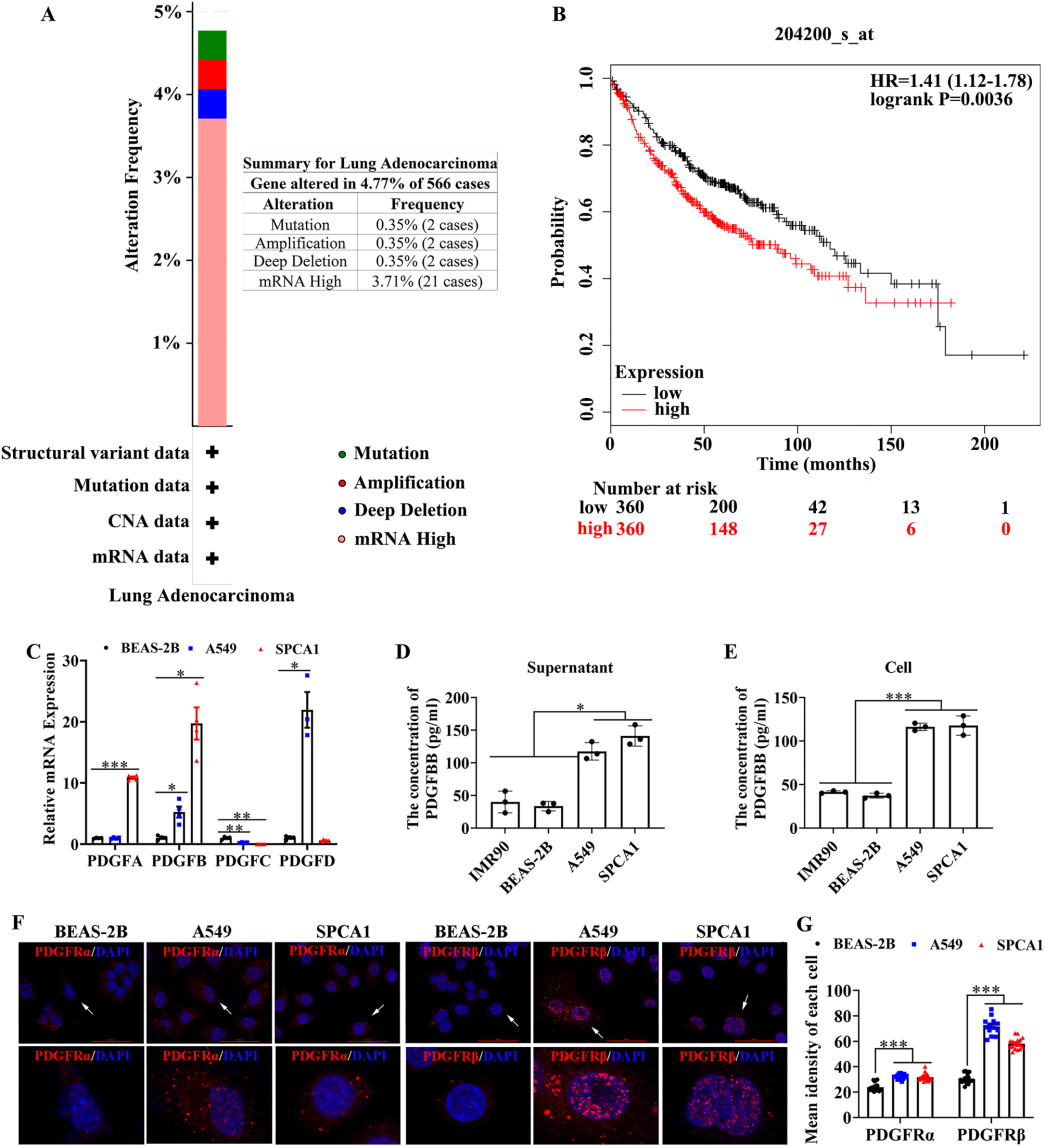
The PDGFBB expression and the Kaplan–Meier analysis in LUAD. (**A**) The frequency of gene alteration in lung adenocarcinoma (n = 566). (**B**) The Kaplan–Meier analysis of the LUAD patients with high or low *PDGFB* expression (n = 566). (**C**) The mRNA level of *PDGFA*, *B*, *C* and *D* in BEAS-2B, A549 and SPCA1 cells (n = 3–4). (**D, E**) The PDGFBB protein level in the supernatant and the cell of BEAS-2B, A549 and SPCA1 cells (n = 3). (**F, G**) The expression and distribution of PDGFRα and PDGFRβ in BEAS-2B, A549 and SPCA1 cells (n = 13–20). The lower panel was an enlarged view of the cells indicated by the arrows in the upper panel. BEAS-2B: a normal human lung epithelial cell; A549 and SPCA1 cells: lung adenocarcinoma cells. *P < 0.05, **P < 0.01, ***P < 0.001 by one-way ANOVA (**C, D, E, G**).

By Cbioportal online tool, we found that of the 566 samples, the frequency of mutation, amplification, deep deletion and mRNA high of *PDGFB* accounted for 0.35% (2 cases), 0.35% (2 cases), 0.35% (2 cases) and 3.71% (21 cases), respectively (see Fig. 1A, Fig. S1, Table S2). Obviously, the proportion of mRNA high of *PDGFB* was the highest, with 3.71% in the population of LUAD (Fig. 1A, Fig. S1, Table S2). Furthermore, the survival probability of the LUAD patients with high expression of *PDGFB* were lower than that of the patients with low expression of *PDGFB* (P = 0.0036, Fig. 1B). The median survival time in high *PDGFB* expression cohort was 89 months, which was shorter than 117.33 months in low *PDGFB* expression cohort. In addition, our data revealed that the mRNA level of *PDGFB* was up-regulated in both A549 and SPCA1 cells (two lung adenocarcinoma cell lines) in comparison with the normal lung epithelial cell (BEAS-2B), while the mRNA expression of *PDGFA, C* and* D* was not always highly expressed in A549 and SPCA1 cells (Fig. 1C). As expected, the protein level of PDGFBB also increased in the supernatant and intracellular part of A549 and SPCA1 cells (Fig. 1D,E). More importantly, the level of PDGFRα and PDGFRβ, located at nucleus, plasma membrane and cytosol, was higher in A549 and SPCA1 cells than that in BEAS-2B cells, especially the PDGFRβ level (Fig. 1F,G). Based on these results, we believed that PDGFBB harbored an essential role in the progression of lung adenocarcinoma.

### PDGFBB knockdown hampers the proliferation and migration of LUAD cells in vitro

**Figure 2:**
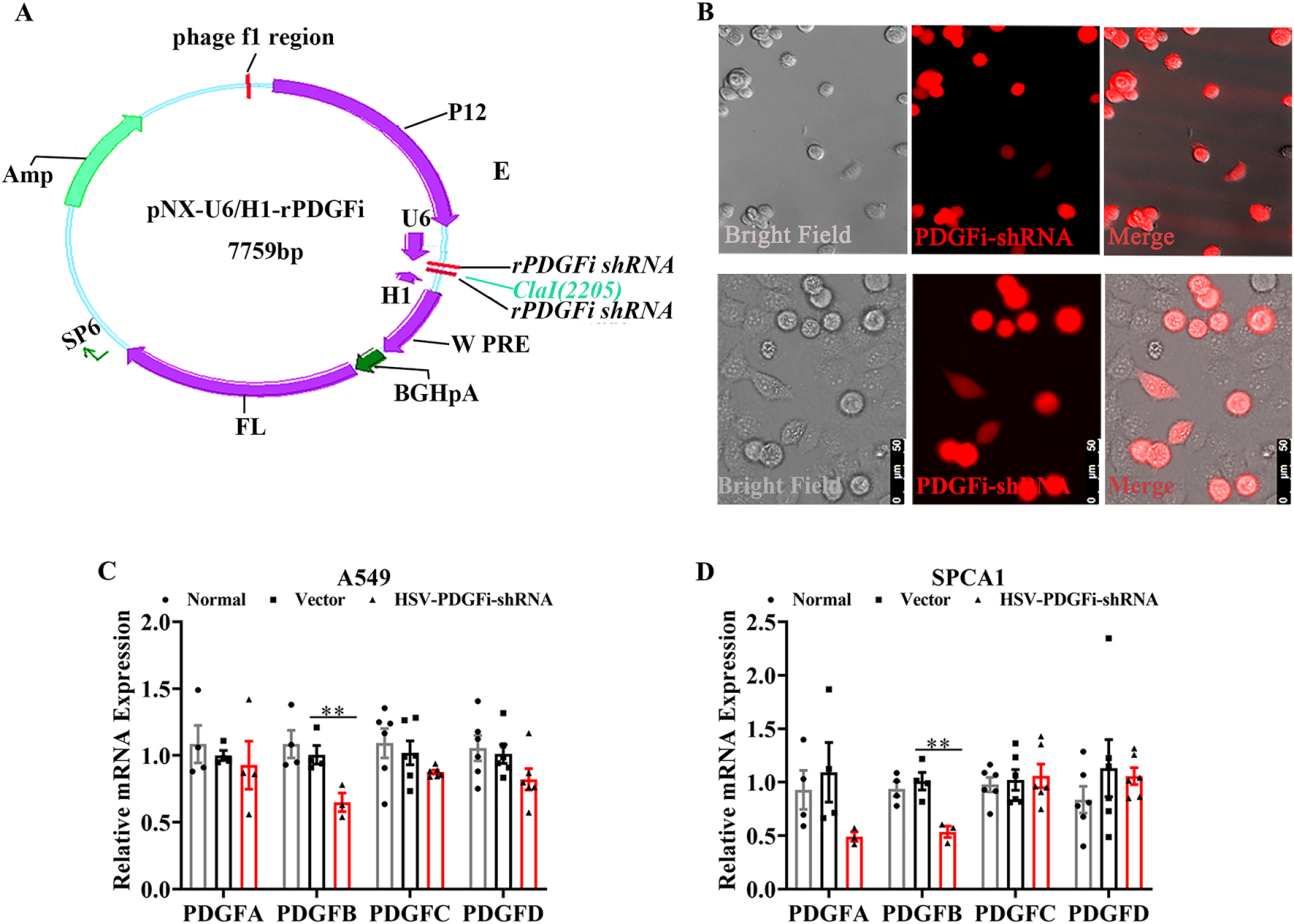
HSV carrying pNX-U6/H1-PDGFi-shRNA recombinant induced the downregulation of *PDGFB* in A549 and SPCA1 cells. (**A**) The atlas of pNX-U6/H1-PDGFi-shRNA construct. (**B**) Fluorescence images showed that after 48 h, the HSV-pNX-U6/H1-shRNA could be transfected into A549 (upper) and SPCA1 (below) cells. (**C, D**) HSV-pNX-U6/H1-PDGFi-shRNA could significantly downregulate the *PDGFB* expression, but *PDGFA*, *C* and *D* in A549 and SPCA1 cells (n = 3–6). **P < 0.01 by one-way ANOVA (**C, D**).

To determine the oncogenic properties of PDGFBB in LUAD, we first cloned pNX-U6/H1-rPDGFi shRNA into herpes simplex virus (HSV) and constructed HSV-PDGFi-shRNA recombinant (shown in Fig. 2A) to study the outcome of LUAD cells by knocking down PDGFBB expression. As shown in Fig. 2, the HSV-PDGFi-shRNA recombinant was constructed successfully, which could be transfected into A549 and SPCA1 cells and downregulate the *PDGFB* expression, but *PDGFA, C* and *D*, in both cells significantly (Fig. 2B–D).

**Figure 3:**
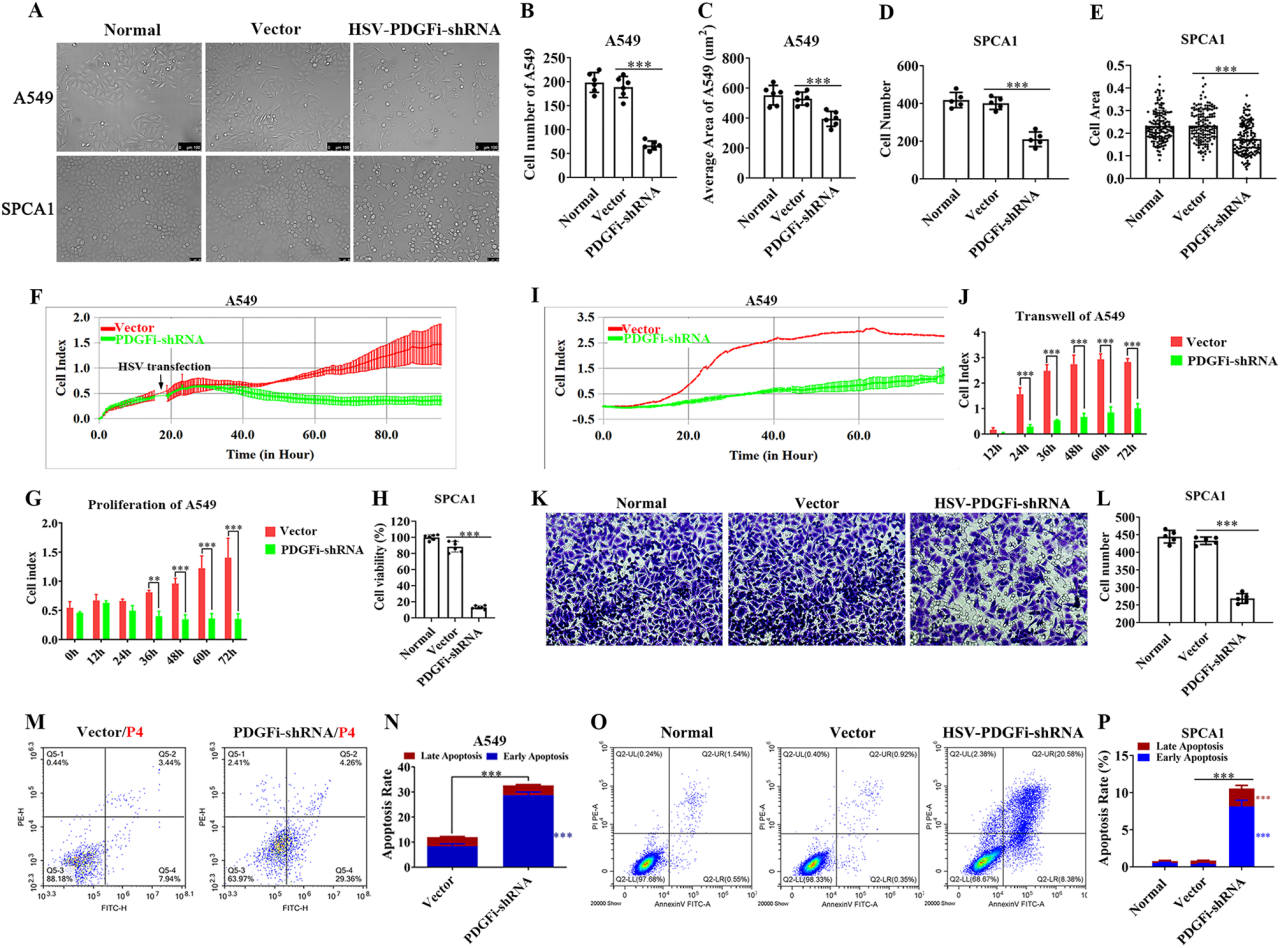
PDGFBB knockdown suppressed the proliferation and migration and induced the apoptosis of A549 and SPCA1 cells in vitro. (**A**) Bright field pictures showed the growth state of A549 and SPCA1 cells after HSV transfection for 48 h. (**B–E**) The cell number and cell area of A549 (n = 6) and SPCA1 (n = 5) cells after HSV transfection for 48 h. The cells in the normal group were the A549 or SPCA1 cells without any intervention. (**F**) After A549 cells were seeded, the cell index for proliferation of A549 was recorded at an interval of 15 min (n = 3). (**G**) The cell index for proliferation of A549 cells after HSV transfection for 0 h, 12 h, 24 h, 36 h, 48 h, 60 h and 72 h (n = 3). Cell index: The relative impedance change of the microelectronic sensor that was integrated into the bottom of the well. (**H**) The cell viability of SPCA1 cells after transfection for 48 h. (**I**) The cell index for migration of A549 cells after HSV transfection for 48 h was recorded at an interval of 15 min (n = 3). (**J**) The cell index for migration of A549 cells after HSV transfection for 12 h, 24 h, 36 h, 48 h, 60 h and 72 h (n = 3). (**K, L**) Transwell assay showed the migration and invasion ability of SPCA1 cells (n = 5). (**M–P**) The apoptosis of A549 and SPCA1 cells after HSV transfection for 48 h (n = 3–4). PDGFi-shRNA: HSV-PDGFi-shRNA. ***P < 0.001 by one-way ANOVA (**B–E, H, L, P**), two-way ANOVA (**G, J**) or/and student t-test (**N**).

After HSV vector and HSV-PDGFi-shRNA were transfected into A549 and SPCA1 cells for 48 h (h), we found that knockdown of endogenous *PDGFB* led to a significant decrease in the cell number and cell area of A549 cells (P < 0.05, Fig. 3A–E), which, however, was unchanged between vector group and normal group (Fig. 3A–E). In addition, the proliferative activity of A549 and SPCA1 cells was also monitored in real time by xCELLigence Real-Time Cell Analyzer and CCK8, respectively. As mentioned in Fig. 3F,G, the cell index curves between the vector group and HSV-PDGFi-shRNA group basically coincided before HSV transfection. However, after HSV transfection for about 24 h, the cell index in HSV-PDGFi-shRNA group started to decrease in a time-dependent manner compared with vector group, and the difference after HSV transfection for 36 h, 48 h, 60 h and 72 h was significant (P < 0.01, Fig. 3F,G). Additionally, the cell viability of SPCA1 cells was also restrained by PDGFBB knockdown (Fig. 3H).

The migratory and invasive ability of A549 and SPCA1 cells was also detected. After A549 cells were transfected with vector or HSV-PDGFi-shRNA for 48 h, the cells were collected and inoculated into CIM plates 16. We found that knockdown of endogenous PDGFBB resulted in the reduced cell index for migration of A549 cells (Fig. 3I,J). What’s more, at 24 h, 36 h, 48 h, 60 h and 72 h, the difference between vector group and HSV-PDGFi-shRNA group was statistically significant (P < 0.001) (Fig. 3J). Likewise, transwell assay showed that the migratory and invasive ability of SPCA1 cells was blocked by PDGFBB knockdown (Fig. 3K,L).

As expected, our results demonstrated that PDGFBB knockdown could trigger the apoptosis of A549 and SPCA1 cells in comparison with vector group, and the difference between two groups was significant (P < 0.001, Fig. 3M–P). Moreover, HSV-PDGFi-shRNA mainly promoted the early apoptosis of A549 and SPCA1 cells, but late apoptosis (that is necrosis) (Fig. 3M–P).

### Exogenous PDGFBB facilitates the growth of A549 and SPCA1 cells in vitro

**Figure 4:**
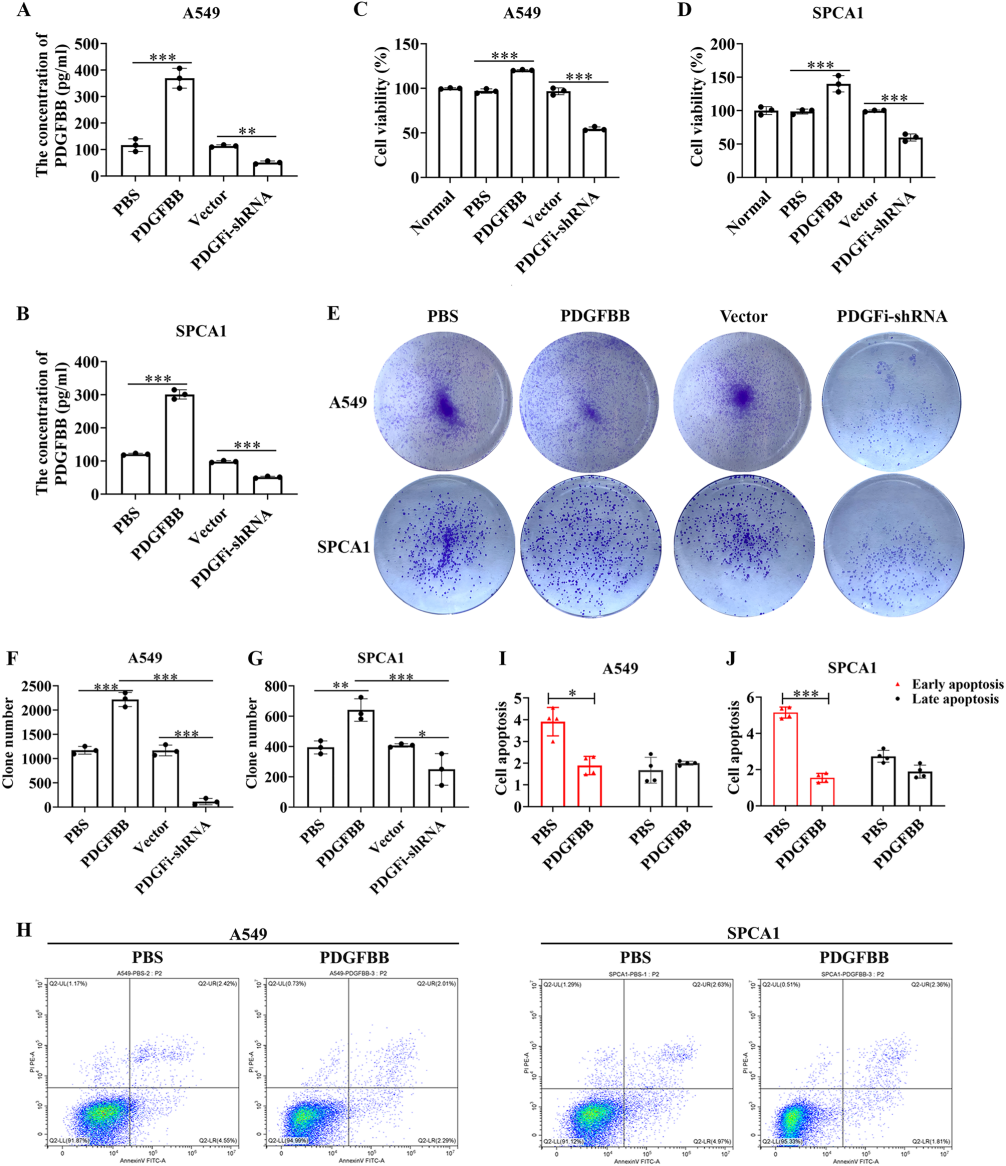
Exogenous PDGFBB boosted the proliferation, but repressed the apoptosis of A549 and SPCA1 cells in vitro. (**A, B**) The PDGFBB protein level in A549 and SPCA1 cells after administrated with HSV-PDGFi-shRNA and exogenous PDGFBB (n = 3). (**C, D**) The cell viability of A549 and SPCA1 cells after administrated with HSV-PDGFi-shRNA and exogenous PDGFBB for 48 h (n = 3). (**E–G**) The number of clones formed by A549 and SPCA1 cells administrated with HSV-PDGFi-shRNA and exogenous PDGFBB (n = 3). (**H–J**) The early and late apoptosis of A549 and SPCA1 cells intervened by exogenous PDGFBB for 48 h (n = 4). *P < 0.05, **P < 0.01, ***P < 0.001 by one-way ANOVA (**A–D, F, G**) or/and two-way ANOVA (**I, J**).

As shown in Fig. 4A,B, HSV-PDGFi-shRNA induced the downregulation of PDGFBB, while it was enhanced in A549 and SPCA1 cells when administrated with exogenous PDGFBB protein (20 ng/ml). Further, HSV-PDGFi-shRNA suppressed the cell viability of A549 and SPCA1 cells, but exogenous PDGFBB could improve the cell viability of both cells (Fig. 4C,D). Similarly, HSV-PDGFi-shRNA led to difficulty in cell clone formation of A549 and SPCA1 cells, while exogenous PDGFBB accelerated the clone formation of both cells (Fig. 4E–G). However, exogenous PDGFBB repressed the apoptosis of A549 and SPCA1 cells (Fig. 4H–J). Likewise, exogenous PDGFBB mainly inhibited the early apoptosis of A549 and SPCA1 cells, but the late apoptosis (Fig. 4H–J).

### A549 cells with PDGFBB knockdown had a low probability of tumorigenesis in vivo

**Figure 5:**
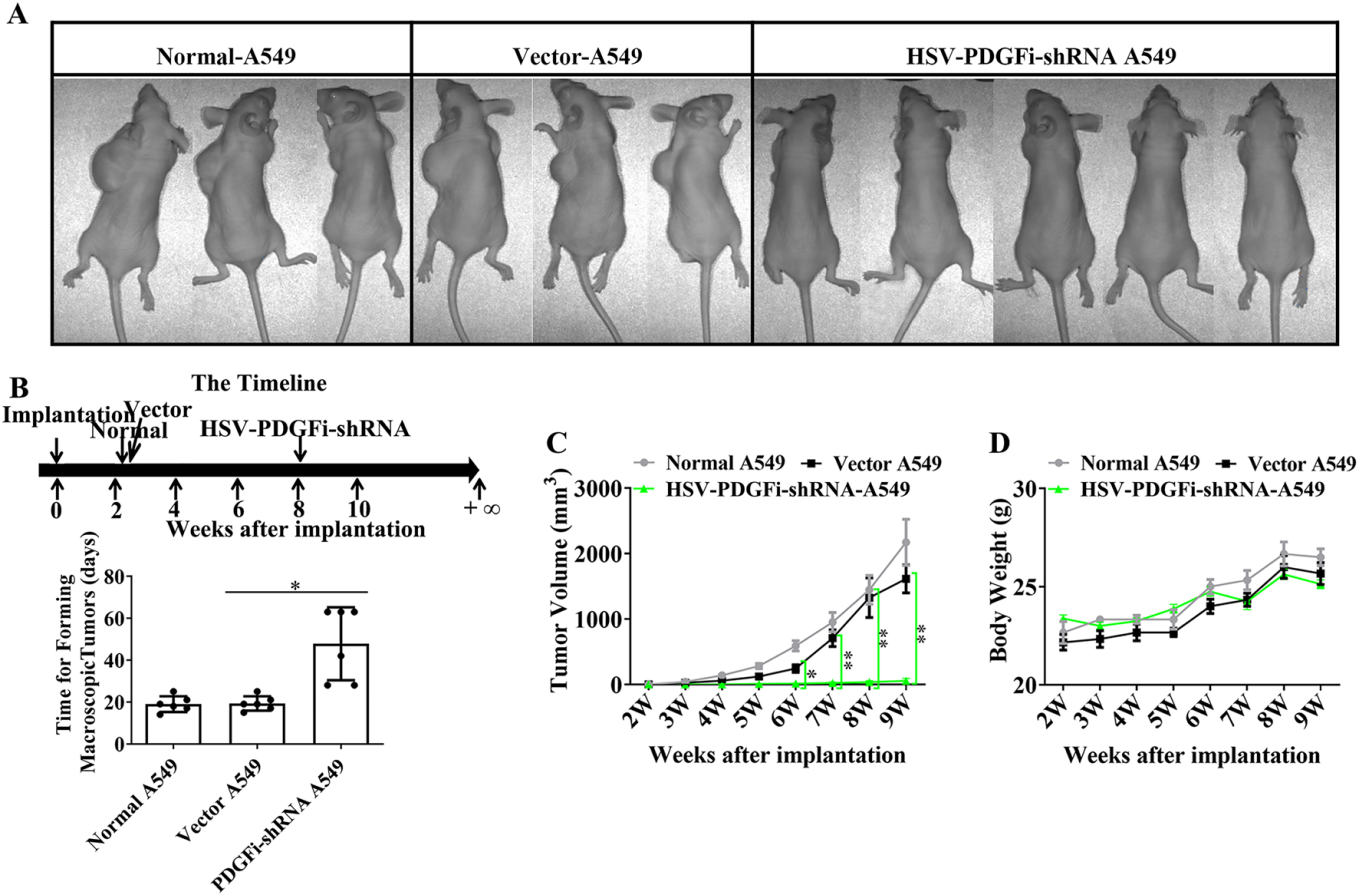
A549 cells transfected with HSV-PDGFi-shRNA had a low probability of tumorigenesis in vivo. (**A**) The representative images showed the tumorigenesis of nude mice in normal-A549 group, vector-A549 group and HSV-PDGFi-shRNA A549 group. (**B**) The time for normal-A549, vector-A549 and HSV-PDGFi-shRNA A549 to form a visible tumor. (**C**) The tumor volume from 0 to 9 weeks after A549 cell inoculation. (**D**) The body weight of nude mice from 0 to 9 weeks after A549 cells inoculation. n = 6–8. *HSV-PDGFi-shRNA group vs vector group, *P < 0.05, **P < 0.01 by one-way ANOVA (**B**) or/and two-way ANOVA (**C, D**).

The normal A549 cells, A549 cells transfected with vector and A549 cells with PDGFBB knockdown were collected and transplanted subcutaneously on the back of nude mice. They were divided into normal-A549 group, vector-A549 group and HSV-PDGFi-shRNA A549 group. Our data demonstrated that the time to form a visible tumor in normal-A549 group, vector-A549 group and HSV-PDGFi-shRNA A549 group was (18 ± 3.60555) days, (19.6667 ± 1.15470) days and (49 ± 19.17029) days, respectively (Fig. 5B). The time for normal-A549 cells and vector-A549 cells to form a visible tumor was similar (Fig. 5B), indicating that HSV did not affect the cell viability of A549 cells. However, the time for HSV-PDGFi-shRNA A549 cells was far longer than that for vector-A549 cells, and even there was no visible tumor in the injection point of HSV-PDGFi-shRNA A549 cells (Fig. 5A,B). If no tumor was observed, the time to form a visible tumor was recorded as the longest observation time, that is, 63 days (9 weeks).

Due to the very long tumorigenesis time and low tumor formation rate, it was not hard to understand that the tumor volume in HSV-PDGFi-shRNA A549 group was smaller than that in normal-A549 group and vector-A549 group (Fig. 5A,C). Nevertheless, there was no significant difference in the body weight of nude mice among these three groups, suggesting that the growth of nude mice was unaffected by the xenografts (Fig. 5D).

### PDGFBB knockdown restrained the growth of xenografts in vivo

**Figure 6:**
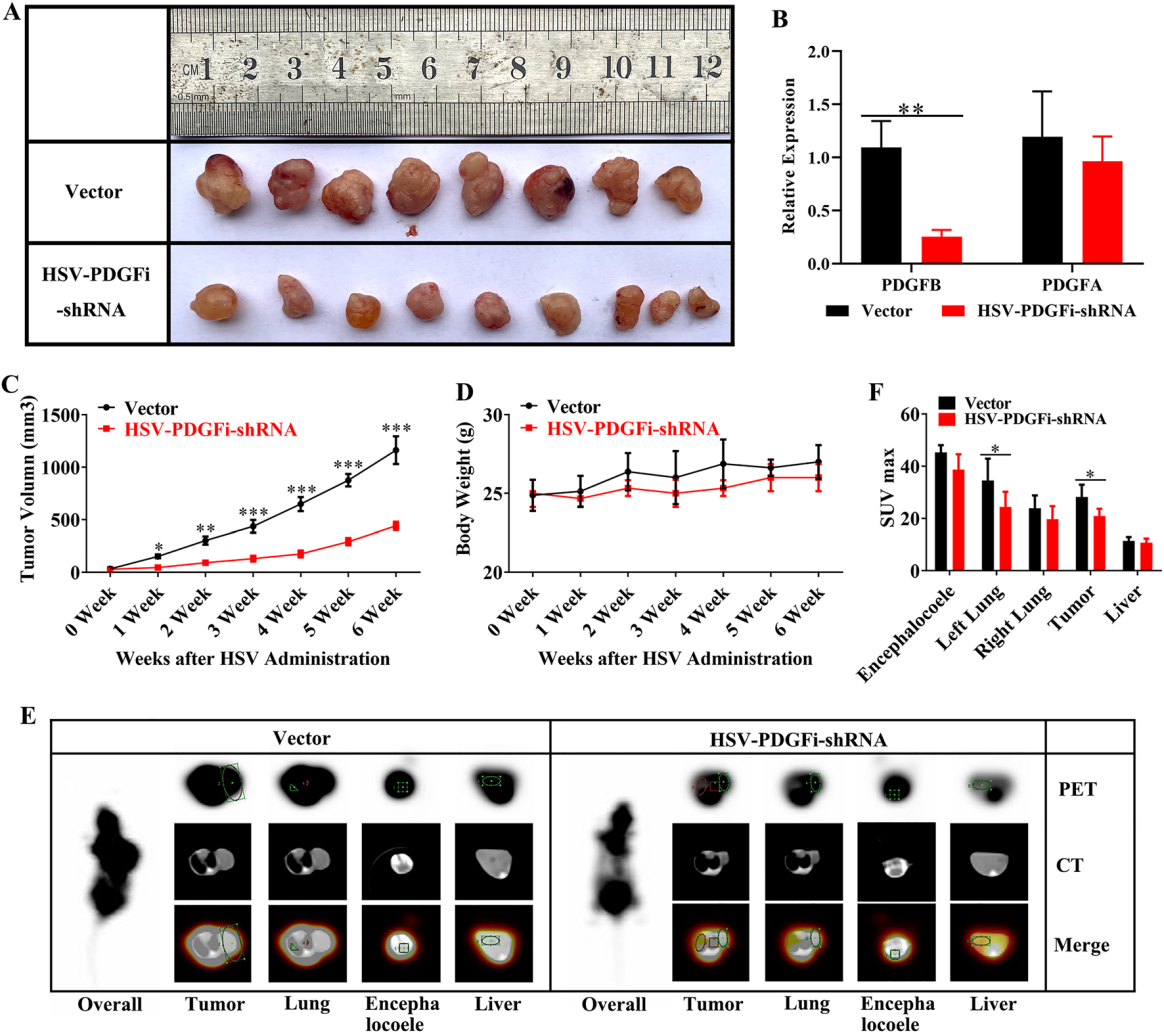
Knockdown of PDGFBB restrained the growth of xenografts in vivo. (**A**) The obtained xenografts after HSV administration for 6 weeks (n = 8–9). (**B**) The *PDGFB* and *PDGFA* expression in xenografts after HSV administration for 6 weeks (n = 6). (**C**) The tumor volume from 0 to 6 weeks of HSV administration (n = 8–9). (**D**) The body weight of nude mice from 0 to 6 weeks of HSV administration (n = 8–9). (**E**) PETCT images showed the cross sections of xenograft, lung, encephalocoele and liver in nude mice (n = 3). (**F**) The SUXmax in the xenograft, lung, encephalocoele and liver after HSV administration for 6 weeks (n = 3). *HSV-PDGFi-shRNA group vs vector group, *P < 0.05, **P < 0.01 by student t-test (**B, F**) or/and two-way ANOVA (**C, D**).

To further explore the antitumor characteristics of PDGFBB knockdown in vivo, the normal A549 cells were used for xenotransplantation. After the formed tumors were visible, HSV-PDGFi-shRNA or vector was microinjected into the tumors once every three days for 6 weeks. Our results indicated that HSV-PDGFi-shRNA could significantly downregulate the *PDGFB* level in the xenografts, but *PDGFA* level (Fig. 6B), and PDGFBB knockdown effectively inhibited tumor growth (Fig. 6A,C). However, there was no significant difference in the body weight of nude mice between vector group and HSV-PDGFi-shRNA group (Fig. 6D).

Using the positron nuclide labelled glucose as imaging agent, positron emission tomography computed tomography (PET/CT) could reflect the metabolic change through the amount of the imaging agent uptake. Therefore, the metabolism and metastasis of xenografts were evaluated by PET/CT. The results pointed out that the metabolic index-SUXmax of xenografts in HSV-PDGFi-shRNA group was significantly lower than that in vector group, demonstrating that PDGFBB knockdown could repress the metabolism of tumor in vivo (Fig. 6E,F). In addition, the SUXmax in the right lung, encephalocoele and liver between the two groups was similar, illustrating that the xenografts did not metastasize into the right lung, brain and liver (Fig. 6E,F). Nevertheless, the SUXmax of left lung in HSV-PDGFi-shRNA group was smaller than that in vector group (Fig. 6E,F), which might be caused by the metastatic tumor or the interference of left subcutaneous tumor.

### PI3K/AKT signaling and Ras/MAPK signaling might be the functional downstream nodes for PDGFBB

**Figure 7:**
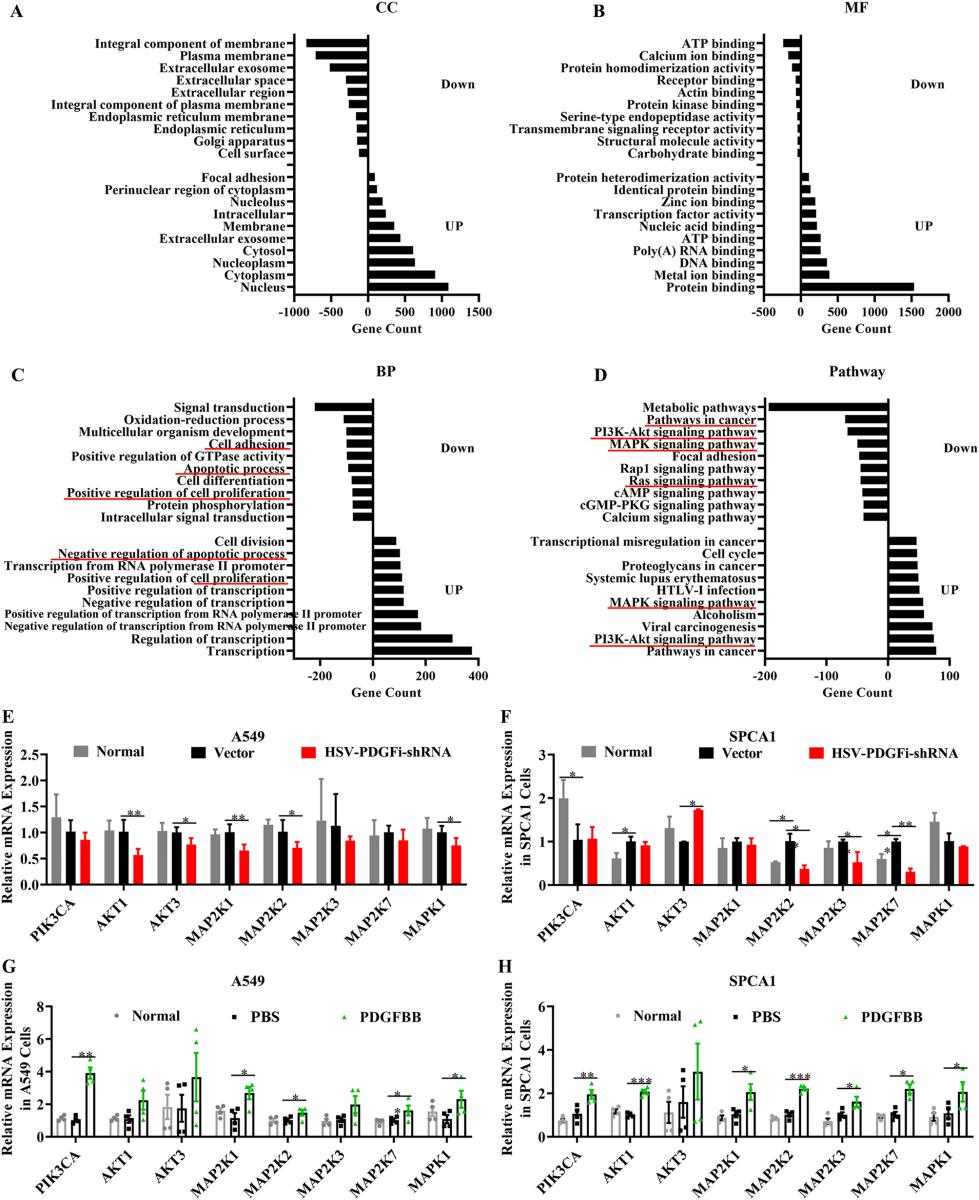
PI3K/AKT signaling and Ras/MAPK signaling might be the functional downstream nodes for PDGFBB. (**A**) The top 10 cellular components of up-regulated DEGs and down-regulated DEGs. (**B**) The top 10 molecular functions of up-regulated DEGs and down-regulated DEGs. (**C**) The top 10 biological processes of up-regulated DEGs and down-regulated DEGs. (**D**) The top 10 enriched KEGG pathways of up-regulated DEGs and down-regulated DEGs. (**E, F**) The key gene expression of PI3K/AKT signaling and Ras/MAPK signaling in the A549 and SPCA1 cells transfected with HSV-PDGFi-shRNA for 48 h by RT-PCR (n = 4). (**G, H**) The key gene expression of PI3K/AKT signaling and Ras/MAPK signaling in the A549 and SPCA1 cells administrated with exogenous PDGFBB for 48 h by RT-PCR (n = 4). *P < 0.05, **P < 0.01, ***P < 0.001 by one-way ANOVA (**E–H**).

**Figure 8:**
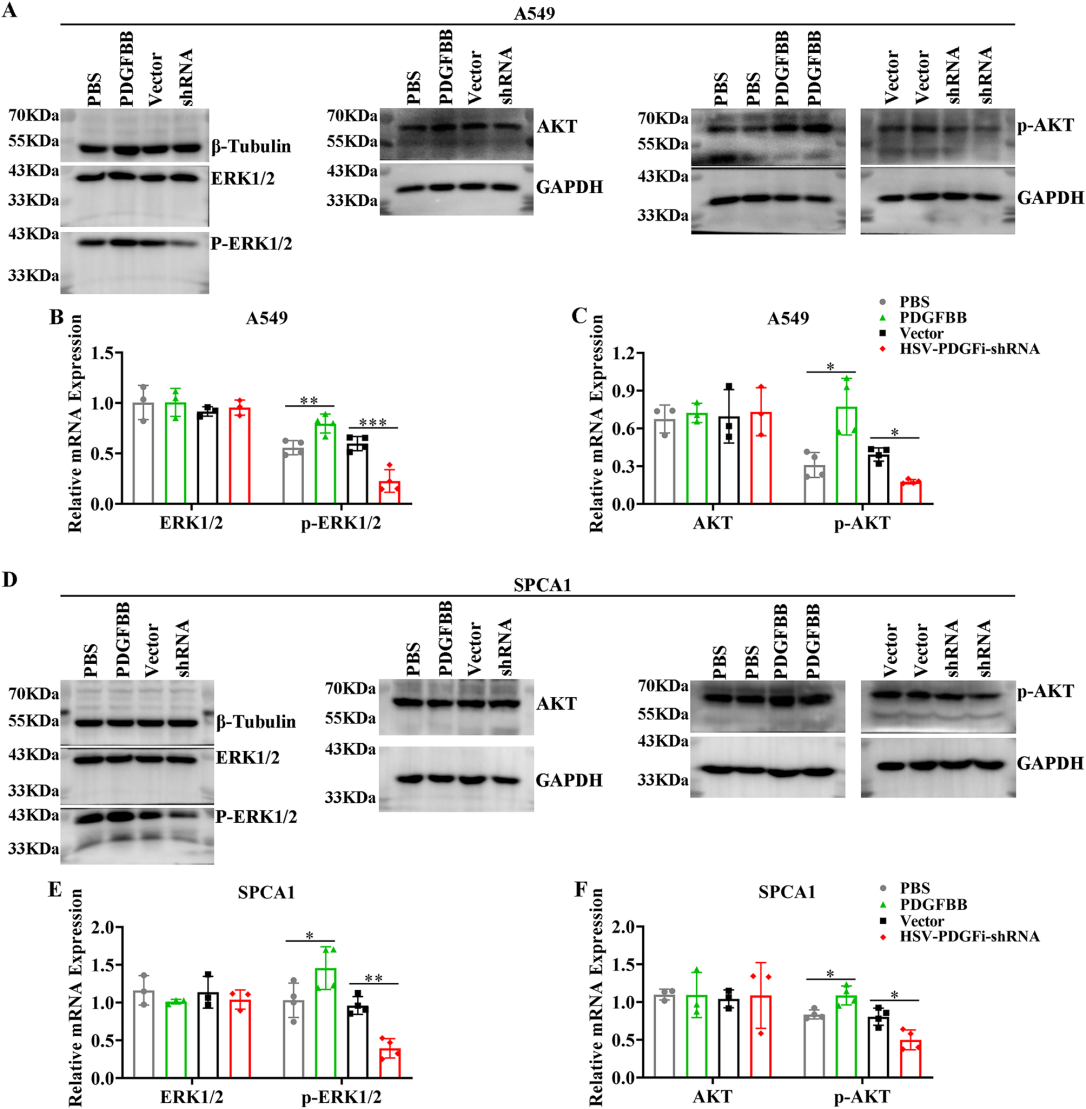
PDGFBB induced the activation of PI3K/AKT and MAPK signaling. The protein expression of ERK1/2, p-ERK1/2, AKT and p-AKT in A549 (**A–C**) and SPCA1 (**D–F**) cells after administrated with exogenous PDGFBB (20 ng/ml) or transfected with HSV-PDGFi shRNA for 48 h. shRNA: HSV-PDGFi shRNA. *P < 0.05, **P < 0.01, ***P < 0.001 by one-way ANOVA (**B, C, E, F**).

Relative to the vector group, there were 7680 differentially expressed genes (DEGs) in the A549 cells transfected with HSV-PDGFi-shRNA, in which 3995 DEGs were upregulated and 3685 DEGs were downregulated (see Table S3). The functional and pathway enrichment analysis of these 7680 DEGs were generated by the Gene Ontology (including molecular function, biological process and cellular component) and KEGG pathway analysis. For all the DEGs, they were distributed in various cellular components and held a wide range of molecular functions (Fig. 7A,B, Tables S4, S5). Moreover, both the up-regulated and down-regulated DEGs were involved in biological processes, including regulation of cell proliferation and regulation of apoptotic process (Fig. 7C, Tables S4, S5), which could determine the fate of tumors. Additionally, both the up-regulated and down-regulated DEGs were enriched in pathways in cancer, PI3K/AKT signaling pathway and Ras/MAPK signaling pathway that were essential for tumor growth and survival (Fig. 7D, Tables S4, S5). Based on the above, we speculated that PI3K/AKT signaling and Ras/MAPK signaling might be the functional downstream nodes for PDGFBB. Therefore, we then detected the key gene expression of these two pathways, including PI3K, AKT, MEK1, MEK2, ERK1 and ERK2 in the A549 and SPCA1 cells transfected with HSV-PDGFi-shRNA or administrated with exogenous PDGFBB. As shown in Fig. 7E, the mRNA expression of AKT1, AKT3, MAP2K1, MAP2K2 and MAPK1 in the A549 cells transfected with HSV-PDGFi-shRNA was significantly reduced in comparison with the vector group. The mRNA level of MAP2K2, MAP2K3 and MAP2K7 was downregulated in SPCA1 cells with PDGFBB knockdown (Fig. 7F). However, exogenous PDGFBB boosted the key gene expression of PI3K/AKT signaling and Ras/MAPK signaling (Fig. 7G,H). In A549 cells, PIK3CA, MAP2K1, MAP2K2, MAP2K3, MAP2K7 and MAPK1 were upregulated by exogenous PDGFBB (Fig. 7G). In the SPCA1 cells, PIK3CA, AKT1, MAP2K1, MAP2K2, MAP2K3, MAP2K7 and MAPK1 were also increased by exogenous PDGFBB (Fig. 7H). To further confirm these results, we also detected the protein level of the key molecules of PI3K/AKT and MAPK pathways (including AKT, phosphor-AKT (p-AKT), ERK and p-ERK1/2) by western blotting. As shown in Fig. 8, exogenous PDGFBB induced the activation of PI3K/AKT and MAPK signaling (indicating by upregulation of p-AKT and p-ERK1/2) in A549 and SPCA1 cells, while PDGFBB knockdown held the opposite effect.

In sum, the above results suggested that PDGFBB mediated growth promotion of lung adenocarcinoma cells might be related to PI3K/AKT signaling and Ras/MAPK signaling.

## Discussion

In LUAD, PDGFBB was upregulated and linked with poor outcomes of the patients. To determine the oncogenic properties of PDGFBB in LUAD, we constructed HSV-PDGFi-shRNA recombinant, which could significantly down-regulated *PDGFB* level, but *PDGFA*, *C* and *D*. Our results demonstrated that PDGFBB knockdown suppressed the proliferation and migration, and triggered the apoptosis of A549 and SPCA1 cells in vitro. Rather, the exogenous PDGFBB held the opposite effect. Moreover, the A549 cells with PDGFBB knockdown held a low probability of tumorigenesis and HSV-PDGFi-shRNA restrained the growth of xenografts derived from normal A549 cells in vivo. For further gene expression analysis using microarray, we found that PI3K/AKT signaling and Ras/MAPK signaling might be the functional downstream nodes for PDGFBB.

PDGFs are indirect proangiogenic markers and play an essential role in the tumor growth and metastasis^22–24^. As a critical member of the PDGFs family, PDGFBB was upregulated in many cancers and related to the proliferation, invasion and metastasis. In the dermatofibrosarcoma protuberans, the fusion of the collagen type I alpha 1 (COL1A1) gene with the *PDGFB* gene caused PDGFBB and its receptor (PDGFRβ) autocrine stimulation and cell proliferation^25^. Moreover, PDGFBB and PDGFR-alpha were highly expressed and associated with poor prognosis in non-small cell lung cancer (NSCLC) cells^26,27^. Specifically, PDGFBB overexpression accounted for approximately 70% of the NSCLS population by immunohistochemical staining of NSCLC tissues^28,29^. Besides, PDGFBB-PDGFRβ signaling induced pericyte-fibroblast transition and contributed to tumor invasion and metastasis^21^. However, anti-PDGF drugs exerted significant inhibitory effect on tumor growth and metastasis in high PDGFBB-producing tumors^30^. Kuzmanov A and his partners pointed out that pharmacological inhibition of PDGFBB/PDGFRβ signaling reduced vessel functionality, tumor growth and cell migration and invasion during breast carcinogenesis^20^. As supported by these studies, our results also showed that PDGFBB was upregulated in lung adenocarcinoma, and knockdown of PDGFBB significantly repressed the tumorigenesis and malignancy of LUAD cells in vitro and in vivo, while exogenous PDGFBB possessed a promoting effect.

To further clarify the downstream mechanism of PDGFBB, we analyzed the differentially expressed genes in PDGFBB knockdown and vector groups by gene microarray analysis. The results suggested that PI3K/AKT signaling and Ras/MAPK signaling might be the functional downstream nodes for PDGFBB. As all know, PI3K/AKT signaling and MAPK signaling were dysregulated almost in all cancers and participated in cell proliferation, apoptosis inhibition, cell migration and cell cancerous transformation^31,32^. However, inhibition of the PI3K/AKT/mTOR signaling and MAPK signaling could play a positive role in apoptosis and anti-proliferation^33–36^. In fact, PDGFBB (like the dose (20 ng/ml) used in this study) could not only induce the proliferation of lung cancer cells, but also lead to activation of ERK and AKT signaling as previously reported by Kinoshita et al.^37^. At first, PDGFBB, binding to its receptor-PDGFRβ, induced the cell proliferation and migration through activating the PI3K/AKT/mTOR signaling pathway^38^. Then Tchougounova E and his coworkers found that the PDGFBB-induced activation of the mitogen-activated protein kinase (MAPK) pathway via extracellular signal-regulated kinase was involved in the initiation of low-grade oligodendrogliomas^18^. Furthermore, PDGFBB evoked rat pleural mesothelioma cell proliferation via activated ERK1/2 and p38 MAPK, which, however, be suppressed by downregulation of either ERK1/2 or p38δ MAPK^39^. Therefore, we could believe that PI3K/AKT signaling and Ras/MAPK signaling might be the functional downstream nodes for PDGFBB.

Gene-targeting therapy is a novel field of medicine with a rosy future in treating a variety of human diseases^40,41^. In this study, PDGFBB served as the target and the vector was herpes simplex virus. HSV had large packaging capacity^42^. Since Ann Surg in 1996 found that the hrR3 HSV vector effectively destroyed HT29 human colon carcinoma cells^43^, HSV-based gene therapy aroused broad attention on treating various cancers in recent years^44,45^. HSV vector was applied to deliver antiangiogenic factors, tumor-suppressor genes, prodrug-activating genes and immune-stimulatory genes^46,47^. In addition, our results also revealed that HSV carrying PDGFi-shRNA could effectively knock down the expression of PDGFBB in vitro and in vivo. Therefore, it was reasonable and feasible to select HSV as the vector for gene targeted therapy in this study.

In conclusion, knockdown of endogenous PDGFBB hampered the tumorigenesis and malignancy of lung adenocarcinoma, while the effect of exogenous PDGFBB was converse. Mechanistically, this outcome might be associated with the functional downstream nodes for PDGFBB, that is PI3K/AKT signaling and Ras/MAPK signaling.

## Materials and methods

### Cell culture

Lung adenocarcinoma cells—A549 and SPCA1, and normal lung epithelial cell BEAS-AB cell line were purchased from American type culture collection (USA). Cells were cultured in Dulbecco’s Modified Eagle’s Medium with high glucose (Hyclone, USA), containing 10% (v/v) fetal bovine serum (Gibco, USA), and 100 U/ml penicillin and streptomycin (Hyclone, USA) and maintained in the humid atmosphere with 5% CO_2_ at 37 °C. The 0.25% trypsin (Hyclone, USA) was used for cell digestion.

### Immunofluorescence

As previously reported^48^, cells were fixed with 4% paraformaldehyde for 15 minutes (min) at room temperature. After rinsing, cells were incubated with 5% sheep serum (Invitrogen, Waltham, MA, USA) and 3 ‰ Triton X-100 at 37 °C for 30 min to block the antigen and permeabilize the membrane. Then primary antibodies (PDGFRα/PDGFRβ, rabbit, 1:100, Abcam, USA) were added and incubated overnight at 4 °C. Then cells were incubated with goat-anti-rabbit secondary antibody (Cy3, 1:100, Jackson, USA) for 1 h at 37 °C. After rinsing, DAPI was added for nuclear staining. Finally, the fluorescence signal was obtained with NIS elements viewer 5.21 software under a Nikon N-SIM-S confocal microscope (Nikon, Japan), and was analysed by Image J software.

### Construction of HSV recombinant

As previously reported^49^, the herpes simplex virus-1 (HSV) carrying pNX-U6/H1-RFP-shRNA constructs were used to generate control shRNA (Vector) and PDGFi-shRNA expressing constructs. In order to obtain non-replicating and low-toxicity HSV vectors, the ICP27, ICP4 and ICP34.5 in HSV were removed. The pNX-U6/H1 plasmid was used to carry the target PDGFi-shRNA sequence (Table S1). After preparation of pNX-U6/H1-PDGFi-shRNA plasmid, the sequences were identified first by enzyme digestion and electrophoresis detection^49^, and then chip-Seq technology. Next, the pNX-U6/H1-shRNA expressing plasmids were packaged into non-replicating and low-toxicity HSV vectors. Since the HSV carrying pNX-U6/H1-shRNA expressing constructs lost their proliferative ability, the vectors were co-transfected into the OG01 cell line, which expressed the ICP27 and ICP4 proteins required for the replication of the virus. Note that due to HSV-PDGFi-shRNA specifically targeted to the *PDGFB* chain (shown in Fig. 2C,D), this study mainly discussed the role of PDGFBB in LUAD.

### Transfection

When the degree of cell fusion reached 30–40%, the HSV viruses were added (multiplicity of infection (MOI) was 10). At transfection for 48 h, the transfection efficiency and the morphology and number of A549 and SPCA1 cells were observed under a fluorescence microscope (Nikon, Japan), and these cells were used for further study.

### Cell proliferation and migration assay

To monitor the proliferation and migration of A549 cells in real time, the xCELLigence Real-Time Cell Analyzer (RTCA) DP Instrument (Roche Diagnostics GmbH, Germany) was used in this study. The relative impedance change (cell index) of the microelectronic sensor that was integrated into the bottom of the well was used to measure the proliferation and migration of A549 cells. For proliferation assay, A549 cells were seeded in E-plates 16 (Roche Diagnostics GmbH) at 5000 cells/well. HSV-PDGFi-shRNA or vector was added after the cells adhered to the wall overnight. Then, the plates were continuously monitored once every 15 min for 72 h. For the cell migration assay, the migration of A549 cells was assessed using specifically designed CIM-plates 16 (Roche Diagnostics GmbH), in which the upper and lower chambers of each well were space out by an 8-μm microporous membrane containing matrigel. The 10% FBS medium was added in the lower chambers, and the cells were seeded into the upper chambers at 40,000 cells/well in serum-free medium. The CIM-plate 16 was monitored every 15 min for 72 h in total. Data analysis was carried out using RTCA software 1.2 supplied with the instrument.

### Cell viability analysis

As reported before^48^, cell viability was detected by CCK-8 kit (DOJINDO, Japan). Briefly, the cells were seeded in 96-well plates with 3000–5000 cells/well. After 48 h of intervention, 10 μl of CCK-8 reagent was added into each well and incubated at 37 °C for 4 h. The absorbance (OD value) was measured by Multiskan Spectrum Microplate Spectrophotometer (Thermo, USA) at a wavelength of 450 nm. The cell viability was calculated by the formula: inhibition rate = (As − Ab)/(Ac − Ab) × 100%, in which As was the absorption of PDGFi-shRNA or PDGFBB (20 ng/ml; PEPROTECH, New Jersey, USA) wells (experimental groups), Ac was the absorption of vector or PBS wells (control groups), and Ab was the absorption of blank control wells (medium wells containing CCK-8 reagent).

### Apoptosis analysis by flow cytometry

The apoptosis assay of A549 and SPCA1 cells was conducted by Annexin V/FITC and PI kit (BD Biosciences, San José, CA). As mentioned previously^50^, after washing with 0.01 M PBS twice, the cells were stained first with FITC-labeled Annexin-V for 30 min at room temperature and in the dark, and then with PI for 5 min. Finally, the stained cells were immediately tested by flow cytometer (BD Accuri™ C6 Plus). The flow cytometer collected 200,000 to 30,000 cells per test.

### Transwell assay

The migration ability was also detected by transwell assay. Refer to the method^48^, the experimental operation was carried out as follows. The experimental device included an upper chamber (Transwell Chamber (Millipore, Burlington, MA, USA)) and a lower chamber, in which the bottom of the upper chamber was an 8-μm microporous membrane and the lower chambers were the wells of 24-well plates (Corning, USA); 5 × 10^4^ cells were seeded into the upper chambers with serum-free medium, while the complete medium containing 10% serum was added into the lower chambers. After 48 h of migration, the noninvasive cells in the upper chambers were removed, and the invasive cells on the bottom of the upper chambers were fixed with 4% paraformaldehyde for 30 min at room temperature and stained with 0.5% crystal violet for 10 min. After rinsing, the invasive cells were photographed by a microscope and counted for statistical analysis.

### Plate clone formation analysis

At first, the normal and PDGFBB-knockdown A549 and SPCA1 cells were prepared. Then, 1000 wells were inoculated into each well of the 6-well culture plates (Corning, USA). After the cells adhered to the wall overnight, the PDGFBB (20 ng/ml) was administrated in the PDGFBB group, and the cells were cultured for 14 days. Meanwhile, the medium was changed every 3 days. When the experiment terminated, the cells were washed with 0.01 M PBS for 3 times and fixed with 4% paraformaldehyde for 15 min. After that, 0.5% crystal violet was applied for clone staining. Finally, the pictures were acquired with a camera, and the number of clones was counted.

### Enzyme-linked immunosorbent assay (ELISA)

The protein level of PDGFBB in the supernatant and cells were determined by ELISA kits (MEIMIAN, Kete Biological Technology Co., Ltd, Jiangsu, China). As reported before^51^, 25 μl of the sample diluent and 25 μl of a sample were added to the sample wells (the final dilution of the sample was 2 times). Meanwhile, 50 μl of the standards were added into the standards wells. Blank wells didn’t add anything. After that, 100 μl HRP-conjugated reagent was added to each well except the blank wells. The plates were covered, mixed gently and incubated for 1 h at 37 °C. After washing, 50 μl of substrate A and 50 μl of substrate B were added to each well to incubate for 15 min at 37 °C. Then, the reaction was stopped by 50 μl of stop solution. Immediately, the absorbance was measured with a spectrophotometer (Thermo Fisher Scientific Oy Ratastie 2, FI-01620 Vantaa, Finland) at a wavelength of 450 nm. Finally, the standard curves were calculated and then the concentration of PDGFBB was calculated.

### In vivo mouse experiments

All the animal experiments were approved by the Animal Ethics Committee of West China Hospital of Sichuan University (Chengdu, China), and the protocols were performed in accordance with the Animal Research: Reporting of in vivo Experiments (*ARRIVE*) guidelines. Female BALB/c nude mice (weighing approximately 20–25 g) between 4 and 6 weeks of age were purchased from Beijing Institutes for Biological Sciences (China). To assess the antitumor activity of HSV-PDGFi-shRNA, the in vivo study consisted of two experiments.

In the first one experiment, we injected A549 cells (5 × 10^6^/mice) transfected with HSV-PDGFi-shRNA or vector into the left side of the armpits of BALB/c nude mice. Then, whether the injected A549 cells could form a tumor, the time required for tumor formation and the tumor growth were observed for 9 weeks.

In the second one experiment, we directly transplanted normal A549 cells (without transfected with HSV-PDGFi-shRNA or vector) into the left side of the armpits of BALB/c nude mice. After the tumors were visible (about 2 weeks after transplantation), the 2 μl HSV-PDGFi-shRNA or vector (5.6 × 10^9^ pfu/ml) was microinjected into the tumors by a Hamilton syringe with a 33G needle once every three days for 6 weeks and the tumor growth was examined concurrently. Finally, after PET/CT analysis, the nude mice were euthanized and sampled.

The tumor width and length were measured by a slide calliper rule every 7 days. The tumor volume was calculated as (length × width × width)/2. The body weight was also recorded every 7 days.

### PET/CT analysis

PET/CT examination was performed by Discovery 690/elite (GE company, USA). Briefly, the nude mice were injected intraperitoneally with the imaging drug-^18^F deoxyglucose (18F-FDG) of 4.2 MBq/kg, and the imaging was completed within 60 min. During the examination, isoflurane was used to induce and maintain the anesthesia. At first, spiral CT scanning was performed. The parameters were as follows: voltage 120 kV, current 260 mA, pitch 0.561, rotational speed 0.5 s/cycle, layer thickness 3.75 mm, interval 3.75 mm, matrix 512 × 512, FOV 50 cm × 50 cm. Then PET scanning was performed. Each mouse was scanned with two beds, each bed was scanned for 2.5 min, and 47 PET cross-sectional images were obtained by CT attenuation correction and iterative reconstruction. After image acquisition, the ROIs of xenograft, lung, encephalocoele and liver were outlined respectively, and the WB-SUVmax of these regions was measured. WB-SUVmax value was used to reflect the energy metabolism in the above areas.

### Total RNA isolation and reverse transcription-polymerase chain reaction (RT-PCR)

According to the instructions, total RNA was extracted by Trizol reagent (Thermo Fisher Scientific Inc). Then, the extracted RNA was reversely transcribed into cDNA with iScript™ cDNA Synthesis Kit (Bio-Rad, USA). The cDNA was subsequently amplified with ITaq Universal SYBR Green Super mix (Bio-Rad, USA). The primers of all the genes involved in this study were shown in Table S1. The reaction conditions were as follows: 95 °C for 30 seconds (s), 95 °C for 5 s, 60 °C for 30 s, 40 cycles. The threshold cycle (Ct) of each sample was recorded by CFX96 fluorescent PCR instrument (Bio-Rad, USA). The data were analyzed by normalization to β-actin values using the 2^−∆∆Ct^ method.

### Western blot (WB)

Referring to the previous description^48^, the protein from A549 and SCPA1 cells (5 × 10^6^) were obtained. The BCA method was used for protein quantitation. Then the proteins were separated by electrophoresis and transferred to PVDF membranes (Immobilon-P membrane, Millipore, Massachusetts, USA). After blocked with 5% (wt/vol) skimmed milk, the membranes were incubated with primary antibodies (shown in Table S1), and then analyzed by immune blotting of HRP-conjugated secondary antibodies (1:5000, GeneTex, USA). Blotting was visualized by an enhanced chemiluminescent chromogenic substrate (Biosharp, China), and captured by Molecular Imager ChemiDocTM XSR + Gel Imaging System (BIO-Rad, USA).

### Gene microarray analysis

The A549 cells transfected with vector or HSV-PDGFi-shRNA were sent to KangChen Bio-tech for gene microarray analysis. DEGs were obtained by comparison of the signal values between groups based on P < 0.05 by t-test, FDR < 0.05, and fold change (FC) > 2 or < 0.5.

### Bioinformatic analysis

The *PDGFB* expression were obtained from the RNA sequencing data of the TCGA database (The Cancer Genome Atlas) using Cbioportal online tool (https://www.cbioportal.org). The study “Lung Adenocarcinoma (TCGA, PanCancer Atlas), 566 total samples” was selected and the profile of mRNA expression was “mRNA expression z-scores relative to diploid samples (RNA Seq V2 RSEM) with a z-score threshold ± 2”. Then, the Kaplan–Meier was analyzed by Kaplan–Meier Plotter database at http://kmplot.com/analysis/index.php?p=service&cancer=lung.

In addition, the gene function and related high-level genome functional information of DEGs from gene microarray assay were annotated by the Gene Ontology and KEGG pathway analysis, which were acquired by DAVID database.

### Statistical analysis

Experimental data from two independent groups and three independent groups and above were compared by independent T-test and one-way analysis of Variance (ANOVA) followed by LSD or Tamhane’s T2 multiple comparisons tests, respectively. In addition, two-way ANOVA (followed by a Bonferroni post hoc test) was used for analyzing the repeated measurement data. The exact analysis of each data is presented in the corresponding figure legend. Statistical significance was accepted at P < 0.05.

### Supplementary Information

Supplementary Figure S1.

Supplementary Figure S2.

Supplementary Legends.

Supplementary Table S1.

Supplementary Table S2.

Supplementary Table S3.

Supplementary Table S4.

Supplementary Table S5.

## Data Availability

The source data can be obtained by the corresponding author or submitting author (email: hxyscientific@foxmail.com).
