# Supplementary figures and images for: PDGFBB facilitates tumorigenesis and malignancy of lung adenocarcinoma associated with PI3K-AKT/MAPK signaling
Source: Sci Rep. 2024 Feb 20;14:4191. doi: 10.1038/s41598-024-54801-7 (PMC10879171; doi:10.1038/s41598-024-54801-7)

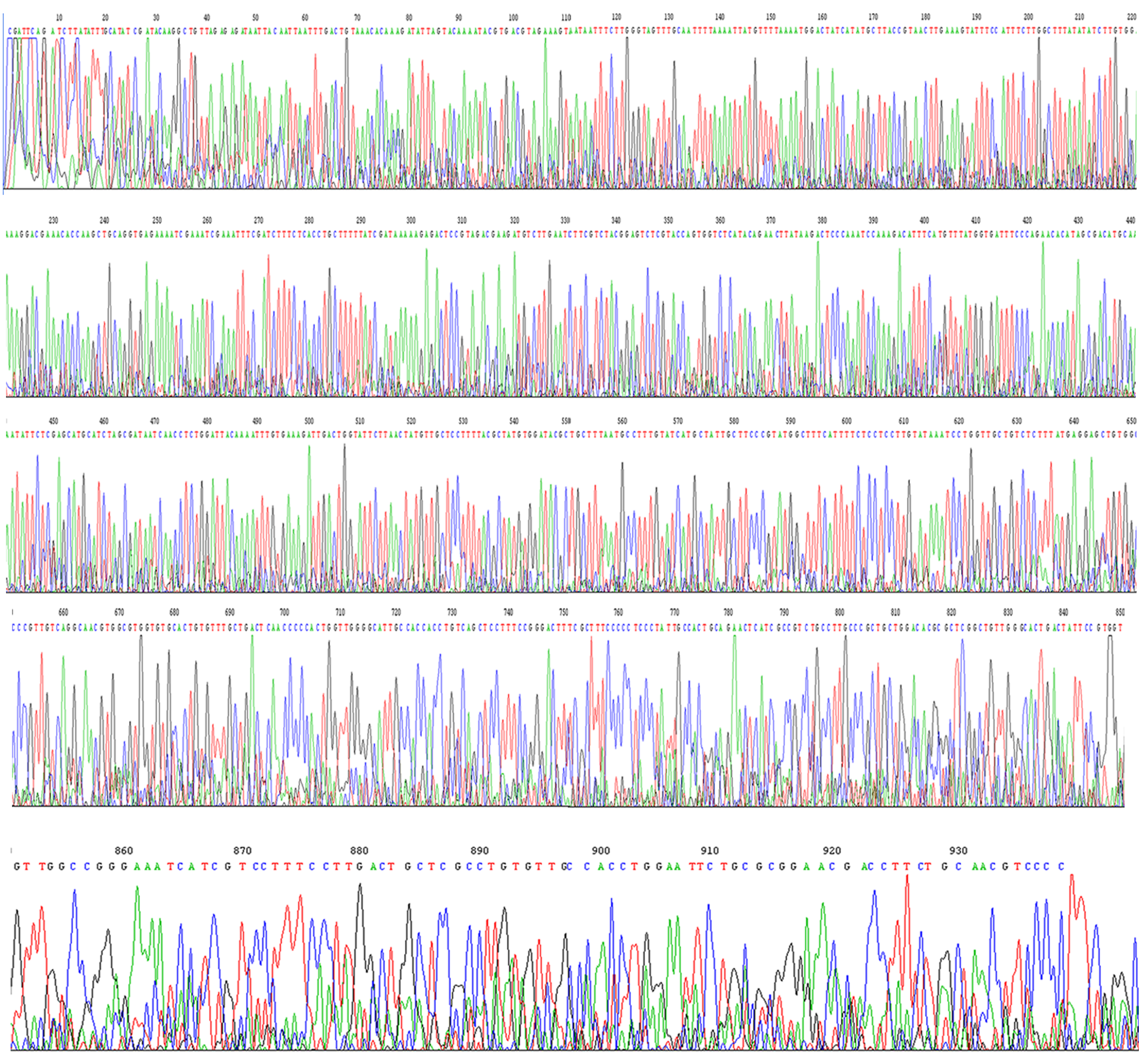

Supplement: Supplementary file 2 — Supplementary Figure S2. [file 41598_2024_54801_MOESM2_ESM.tif]
