# Supplementary material for: PDGFBB facilitates tumorigenesis and malignancy of lung adenocarcinoma associated with PI3K-AKT/MAPK signaling
Source: Sci Rep. 2024 Feb 20;14:4191. doi: 10.1038/s41598-024-54801-7 (PMC10879171; doi:10.1038/s41598-024-54801-7)
Supplement: Supplementary file 3 — Supplementary Legends. [file 41598_2024_54801_MOESM3_ESM.pdf]

PDGFBB Facilitates Tumorigenesis and Malignancy of Lung Adenocarcinoma Associated with PI3K-AKT/MAPK Signaling

He Xiu-Ying[1,2], Zheng Yue-Xiang[4], Yang Hui-si[4], Yu Hong-Zhu[2], Xia Qing-Jie[2*], Wang Ting-Hua[1,2,3*]

1. Department of Anesthesiology, West China Hospital, Sichuan University, Chengdu, China.
2. Institute of Neurological Disease, West China Hospital, Sichuan University, Chengdu, China.
3. Institute of Neuroscience, Laboratory Zoology Department, Kunming Medical University, Kunming, China.
4. School of integrated traditional Chinese and Western medicine, Southwest Medical University, Luzhou, China.

The title of supplementary files

Table S1. The primers for RT-PCR, the target PDGFi-shRNA sequence, and the primary antibodies for WB.
Table S2. Patients data and mRNA expression z-scores relative to diploid samples.
Table S3. Differentially expressed genes with fold change greater than 2.
Table S4. The BP, CC, MF and Pathway of upregulated DEGs.
Table S5. The BP, CC, MF and Pathway of down regulated DEGs.
Fig S1. The frequency of gene alteration in 566 patients with lung adenocarcinoma.
Fig S2. The sequence of pNX-U6/H1-PDGFi-shRNA construct.
